# Attenuation of imidacloprid deleterious effect on hepatic and neural tissues via acetylsalicylic acid: targeting HMGB1/caspase-3 axis and inflammatory pathway

**DOI:** 10.1007/s00210-026-05109-y

**Published:** 2026-03-16

**Authors:** Asmaa M. Ragab, Ahmed A. Mohamed, Walied Abdo, Heba M. Hashem, Mostafa Ali Elmadawy, Ahmed S. Saad, Mohamed Dmerdash, Tohada M. AL-Noshokaty

**Affiliations:** 1 Department of Pesticides Chemistry and Toxicology, Faculty of Agriculture, Kafrelsheikh University, El Geish Street, Kafrelsheikh, 33516 Egypt; 2 Department of Biochemistry, Faculty of Pharmacy, New Mansoura University, New Mansoura, 7723730 Egypt; 3 Department of Pathology, Faculty of Veterinary Medicine, Kafrelsheikh University, El Geish Street, Kafrelsheikh, 33516 Egypt; 4 Department of Pharmacy Practice, Faculty of Pharmacy, Sinai University, Third Al Areesh, 16020 North Sinai Governorate Egypt; 5 Department of Forensic Medicine and Toxicology, Faculty of Veterinary Medicine, Kafrelsheikh University, El Geish Street, Kafrelsheikh, 33516 Egypt; 6 Department of Pharmacology and Toxicology, Faculty of Pharmacy, Port Said University, Port Fouad City, 42526 Egypt; 7 Pharmacology & Toxicology Department, Clinical Pharmacy Program, East Port Said National University, Port Said City, 42526 Egypt; 8 Department of Anatomy, Faculty of Medicine, Al-Azhar University, Cairo, 11884 Egypt; 9 Biochemistry Department, Faculty of Pharmacy, Heliopolis University, Cairo, 11785 Egypt

**Keywords:** Imidacloprid, Hepatoxicity, Neurotoxicity, Acetylsalicylic acid, HMGB1

## Abstract

Imidacloprid (IMID), a neonicotinoid insecticide, is widely utilized but has been implicated in systemic toxicities affecting hepatic, renal, and cerebral tissues. Recent observations indicate concurrent field application of IMID with acetylsalicylic acid (ASA), producing synergistic in plant protection. This study investigates the modulatory impact of ASA on IMID-induced hepatoneurotoxicity in male albino rats. Rats (*n* = 20) were allocated into four experimental groups: control, ASA-treated (40 mg/kg b.w.), IMID-treated (20 mg/kg b.w.), and IMID + ASA combination. Serum biomarkers of hepatic injury such as alanine aminotransferase (ALT), aspartate aminotransferase (AST), albumin, and total protein were estimated alongside brain acetylcholinesterase (AchE) activity. Oxidative stress parameters were assessed by measuring malondialdehyde (MDA) concentrations and antioxidant enzyme activities, specifically superoxide dismutase (SOD) and catalase (CAT), in liver and brain homogenates. Gene expression of pro-inflammatory cytokine, including high mobility group box 1 (HMGB1), tumor necrosis factor-alpha (TNF-α), and interleukin-6 (IL-6), and immunohistochemical expression of nuclear factor kappa B (NF-κB P65) were analyzed. IMID exposure resulted in significant hepatocellular and neuronal damage characterized by elevated serum ALT and AST, hypoalbuminemia, hypoproteinemia, increased MDA, suppression of SOD and CAT activities, and upregulated mRNA expression of TNF-α, IL-6, and HMGB1. Co-administration of ASA markedly mitigated these biochemical and histopathological alterations, normalizing antioxidant defenses and downregulating inflammatory mediators and NF-κB activation. The obtained results suggest that ASA is not toxic in this context and may actually protect against the liver and brain damage caused by IMID by dampening the oxidative stress and inflammatory responses driven by HMGB1 and NF-κB.

## Introduction

Imidacloprid (IMID) is a neonicotinoid insecticide chemically known as 1-(6-chloro-3-pyridylmethyl)-N-nitroimidazolidin-2-ylideneamine. IMID is classified as a pyridylmethyl amine derivative and designated as a Group E chemical by the HED RfD Peer Review Committee (1111 0/93) (Thyssen and Machemer 1999). IMID is an effective pesticide against aphids, cucumber beetles, and whiteflies, including species such as silverleaf and sweet potato whiteflies (Abdel razik et al. 2022). The pesticidal mechanism of action involves disrupting nervous system function by inhibiting nicotinic acetylcholine (Ach) receptors. This inhibition blocks Ach-mediated signaling at the postsynaptic membrane, thereby impairing normal nerve transmission (Cartereau et al. 2021).

IMID-induced acute oral and dermal toxicity classifications for animals are established by the World Health Organization and the U.S. Environmental Protection Agency, with imidacloprid included among these substances. However, overuse of IMID can lead to environmental contamination, posing risks to humans and animals through the food chain. It can also harm non-target pollinators and disrupt the natural ecological balance (Tudi et al. Jan. 2021).

Oxidative stress serves as an effective biomarker for evaluating the harmful effects of insecticides in various organisms (Devi, et al. 2024). Oxidative damage adversely affects biological systems by causing injury to lipids, DNA, and proteins, ultimately leading to cellular apoptosis. Numerous studies have demonstrated that imidacloprid induces oxidative stress and lipid peroxidation in humans and cell cultures (Mahajan et al. 2018; Silva et al. 2022). Experimentally, oral administration of 20 mg/kg IMID to female rats significantly increased malondialdehyde (MDA) levels while decreasing redox biomarkers, including GSH, CAT, and SOD in the liver, kidneys, and brain (Abdelhafez et al. 2023). Moreover, IMID has demonstrated the capacity to induce nephrotoxicity and hepatotoxicity in animal tests, predominantly via oxidative stress and inflammation (Arfat et al. 2014). IMID exposure results in renal impairment, characterized by tubular injury and heightened oxidative biomarkers, as well as hepatic injury, shown by higher blood enzymes, hepatocyte degeneration, and increased liver mass (Arfat et al. 2014; Tang et al. 2025).

IMID toxicity was evident in both animals and humans (Pang et al. 2020). This effect is dose-dependent, marked by the activation of apoptotic pathways such as high mobility group box 1 (HMGB1)/caspase-3 pathway and the impairment of antioxidant status (Chi et al. 2015). Significantly, dosages far below the accepted lethal threshold can provoke substantial biochemical and histological alterations in the kidneys and liver, with some evidence indicating that antioxidant-rich compounds may alleviate these toxic effects (Architha et al. 2023(

Recently, many farmers have combined acetylsalicylic acid (ASA) with IMID due to their complementary phytoprotective properties. ASA, commonly known as aspirin, is derived from salicylic acid, a naturally occurring phenolic compound found in various plants. Salicylic acid plays an essential role in a complex signal transduction network that coordinates plant development, growth, and defense responses. Medically, ASA is used as an analgesic and anti-inflammatory drug and is currently primarily employed as an antiplatelet agent for prophylaxis in cardiac patients (Karolczak et al. 2013). However, some complications have also been observed, such as oxidative stress affecting various tissues, including different epithelial tissues and even nervous tissue. The mechanisms of ASA toxicity include oxidative stress, inhibition of cyclooxygenase enzymes, and mitochondrial uncoupling (Sidlak et al. 2025).

To the best of our knowledge, the toxicological effects of combined exposure to ASA and IMID have not yet been investigated. Therefore, this study aims to evaluate whether ASA can mitigate or exacerbate IMID toxicity in albino rats. This research focuses on inflammatory and antioxidant markers and the cell death receptor pathway, including the HMGB1/caspase-3 pathway, which is associated with IMID toxicity. Specifically, we examined administering sublethal doses of IMID concurrently with ASA, especially regarding oxidative stress and inflammatory pathways.

## Materials and methods

### Chemicals

- IMID (95.45% pure) was acquired from the Central Agricultural Pesticides Laboratory (CAPL), Agricultural Research Center, Dokki, Egypt.
- Acetylsalicylic acid (ASA) (Aspocid® tablets, The Arab Drug Co., Egypt), each tablet that contains 75 mg ASA, was purchased from local pharmacies and administered at a dose of 40 mg/kg in a solution that was recently set within 5 min of administration.

### Experimental animals

In accordance with the “Principles of Laboratory Animal Care” (National Institutes of Health Publication No. 85-23, updated 1985), the animal handling was approved by the Committee of Institutional Animal Care and Use (KFS-IACUC) at Kafr Elsheikh University, Egypt (Number of Approval: KFS-IACUC/277/2025). In addition, all animal experiments were conducted in firm agreement with the ARRIVE guidelines. Twenty albino male rats, weighing between 150 and 180 g, were used. Giza, Egypt’s National Research Center, was the site of specimen acquisition. Each animal that participated in this study was kept in a controlled environment with a temperature of 25 ± 2 °C, a 12-h light/dark cycle, and unlimited access to food and water.

### Experimental design

**Fig. 1:**
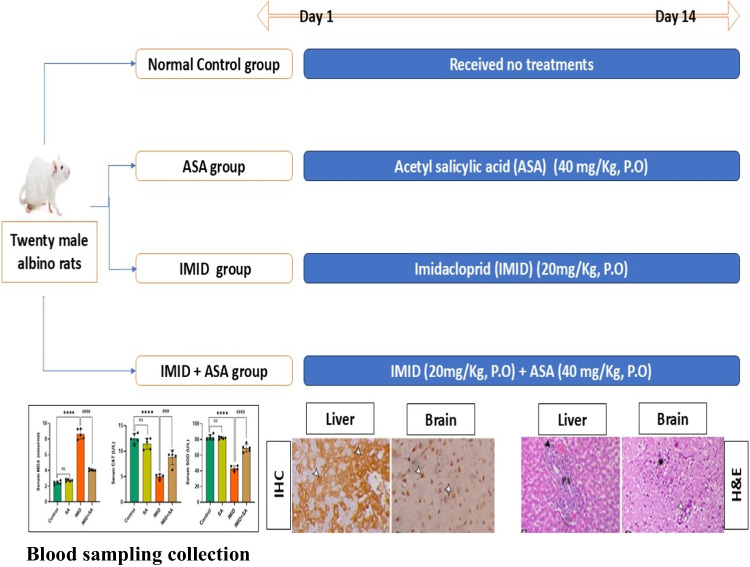
Schematic representation of the experimental design and timeline

Twenty rats were divided into four groups (5 rats/each) (Figure 1).

•Control group: rats received orally distilled water, which was used as a vehicle for chemicals.
•ASA group: rats received acetylsalicylic acid (ASA) 40 mg/kg bw once daily for 14 consecutive days (Biondo-Simões et al. 2021).
•IMID group: rats received imidacloprid (IMID) in saline at a dose level of 20 mg/kg bw equivalent to 1/22 LD50 IMID once daily for 14 consecutive days (Abdelhafez et al. 2023).
•ASA + IMID group: rats received IMID + ASA with the same previously mentioned doses once daily for 14 consecutive days.

### Blood sampling collection

Blood samples were collected, and serum was separated by centrifugation at 3000 rpm for 10 min. The serum was then stored at −20 °C for subsequent biochemical analyses. All animals were euthanized, and the liver and brain were excised and divided into three portions. The first portion was immediately frozen in liquid nitrogen and stored at −80 °C for quantitative real-time polymerase chain reaction (qRT-PCR). The second portion was fixed in buffered formalin (pH 7.2) for histological and immunohistochemical analyses. The third portion was homogenized in ice-cold phosphate-buffered saline (pH 7.4), centrifuged, and the supernatant was stored at −20 °C for further biochemical assays.

### Liver functions assay

Serum ALT and AST activities (Spectrum Diagnostics, Germany), albumin, and total proteins were measured using standard colorimetric methodologies by commercially available kits (Diamond Co., Giza, Egypt).

### Assessment of oxidative stress markers (MDA level, SOD, and CAT activities)

MDA level, SOD, and CAT activities were measured using standard colorimetric methodologies by commercially available kits (Biodiagnostic Company, Dokki, Giza, Egypt).

### Measurement of TNF-α and IL-6 levels in both haptic and brain tissues

The levels of the following markers were determined: IL-6 (Cat# E-HSEL-R0004, Elabscience, Wuhan, China) and TNF-α (Cat# E-EL-R2856, Elabscience, Wuhan, China) according to ELISA manufactures’ kits.

### Measurement of acetylcholinesterase (AchE) activity in the brain tissues

Acetylcholinesterase (AchE) activity was determined by commercially available kits (Cat# E-BC-K174-M, Elabscience, Wuhan, China).

### Histopathological examination of both hepatic and brain tissues

Brain and liver tissues were obtained from all groups, soaked in 10% formol-saline for 24 h, fixed in paraffin wax, sectioned at 4 µm, and stained with hematoxylin and eosin (H&E) (Bancroft and Gamble Jan. 2007). Then, all slides were photographed using a digital camera affixed to an Olympus BX51 optical microscope (Olympus Corporation, Tokyo, Japan).

### Immunohistochemical staining of NF-κB P65 in both hepatic and brain tissues

After the tissue slides were deparaffinized and rehydrated, the rat polyclonal NF-κB P65 antibody (Cat # PA5-27617, Invitrogen, USA) was applied to them overnight at 4 °C. After the slides were washed with PBS, they were incubated with a goat anti-rabbit secondary antibody (Cat# K4003, EnVision+™ System Horseradish Peroxidase Labelled Polymer; Dako) at room temperature for 2 h before being analyzed with the DAB kit. In order to examine the pieces, a light microscope was used. Using the Image J analysis software (NIH, USA), the quantitative assessment of NF-κB immunostaining was performed by calculating the ratio of positive photos.

### Quantitative evaluation of mRNA expression of HMGB1, caspase-3, TNF-α, IL-6, and GAPDH

Trizol reagent (Life Technologies, Camarillo, USA) was used to extract total ribonucleic acid (RNA) from the liver and brain samples. The RNA quantity was assessed using the Maxima SYB Green/Fluorescein qPCR Master Mix (Fermentas, Waltham, MA, USA). One microgram of total RNA was reverse transcribed into single-stranded complementary DNA (cDNA) using the QuantiTect Reverse Transcription Kit (Qiagen, Valencia, CA, USA) following a two-step RT-PCR protocol. The rat GAPDH gene served as the housekeeping gene and internal reference standard.

Table 1 lists the PCR primer sequence of the examined genes. The thermal cycling protocol commenced with an initial incubation at 95 °C for 5 min, succeeded by 40 cycles of 94 °C for 20 s and 60 °C for 1 min. Fluorescence data were collected during the extension phase. Melting curve analysis was conducted to verify the specificity and precision of the PCR data. The Qiagen Rotor-Gene Q (Valencia, CA, USA) autonomously assessed the Ct values and gathered data. The relative expression levels of HMGB1, caspase-3, TNF-α, IL-6, and GAPDH mRNAs were measured by the 2^-DDCt^ methodology.

**Table 1:** Forward (F) and reverse (R) primer sequences of HMGB1, caspase-3, TNF-α, IL-6, and GAPDH

Gene	Primer sequence 5′ → 3′	Sequence accession	Annealing temperature	Cycle numbers	Product size (bp)
HMGB1	F: 5′-GCTGACACCAAGGAGGAAAC-3′ R: 5′-GTTGCAGGCTGGAATCTTCT-3′	NM_012963.4	60 °C	40	180
Caspase-3	**F**: 5′-GGT ATT GAG ACA GAG AGT GG-3′ **R**: 5′-CAT GGG ATC TGT TTC TTT GC-3′	NM_012922.2	60 °C	40	200
TNF-α	F: 5′-ATGGGCTCCCTCTCATCAGTTCC-3′ R: 5′-GCTCCTCCGCTTGGTGGTTTG-3′	NM_001278601.1	60 °C	40	200
IL-6	F: 5′-ACTTCCAGCCAGTTGCCTTCTTG-3′ R: 5′-TGGTCTGTTGTGGGTGGTATCCTC-3′	NM_001314054.1	60 °C	40	220
GAPDH	F: 5′-GAAGGTCGGTGTGAACGGATTTG-3 R: 5′-AATGAAGGGGTCGTTGATGGC-3′	NM_017008	60 °C	40	150

### Statistical analysis

Data are expressed as mean ± standard deviation (SD). A one-way analysis of variance (ANOVA) with Tukey’s post-hoc test was conducted to evaluate quantitative variables utilizing GraphPad Prism version 8 (San Diego, CA, USA). A *p*-value below 0.05 was deemed statistically significant.

## Results

### Hepatoprotective effect of acetylsalicylic acid (ASA) against imidacloprid (IMID)-induced liver injury

**Fig. 2:**
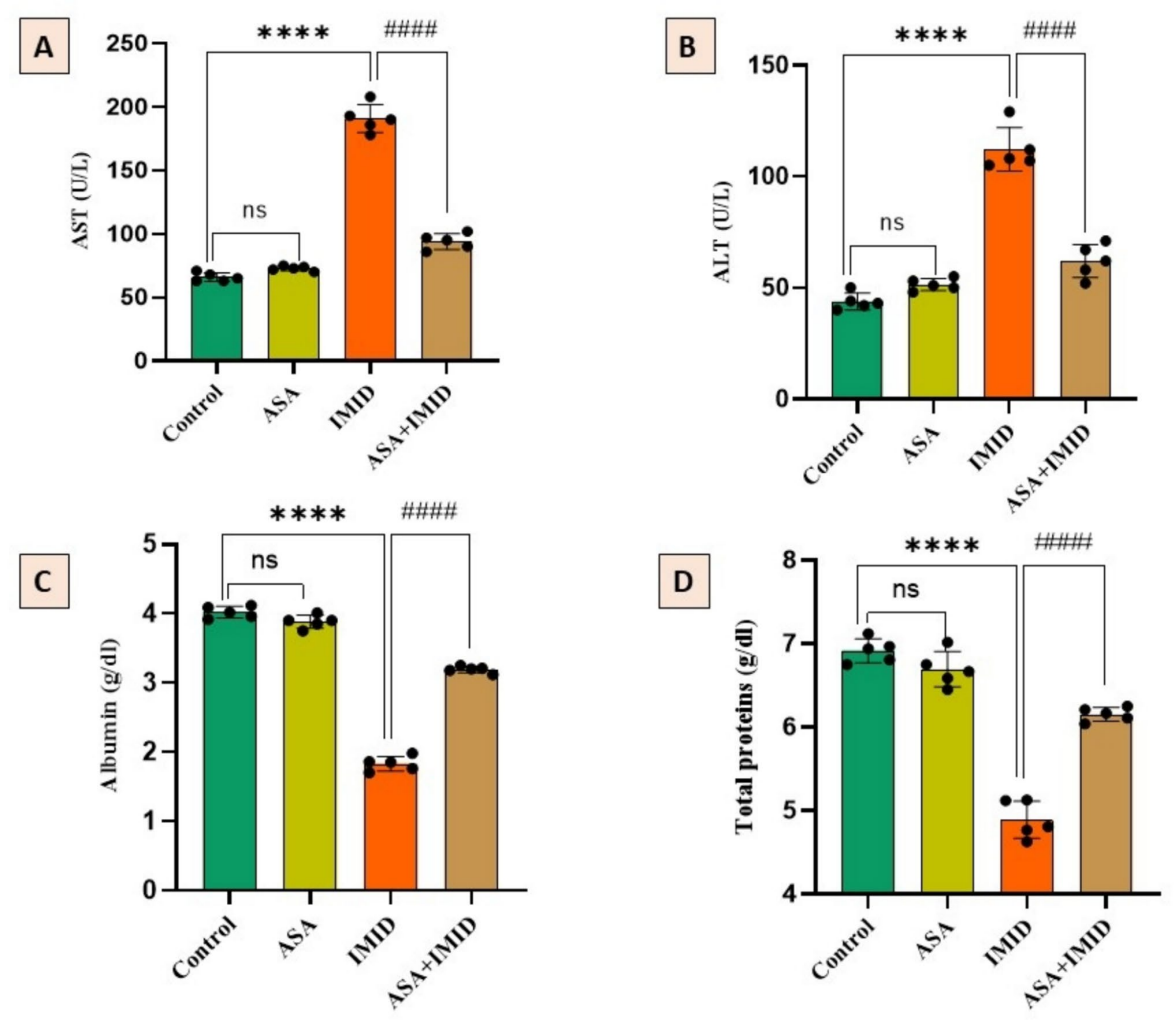
Impact of acetylsalicylic acid (ASA) against imidacloprid (IMID)-induced liver injury. A ALT (U/L), B AST (U/L), **C** albumin (g/dL), and **D** total proteins (g/dL). Values showed as mean ± SD (*n* = 5). Significant difference vs. *normal control (****p < 0.001), ^#^imidacloprid (.^####^*p* < 0.001)

Rats treated with IMID showed hepatic injury shown by a marked increase in serum ALT and AST activities (*p* < 0.001) and decrease in albumin and total proteins levels in comparison with the control group. Interesting, ASA + IMID showed a significant decrease in serum ALT and AST activities and increase in albumin and total proteins levels compared with IMID group (*p* < 0.001). Furthermore, no substantial difference was observed between the control and ASA groups (Figure 2).

### Impact of acetylsalicylic acid (ASA) against imidacloprid (IMID)-induced oxidative stress

**Fig. 3:**
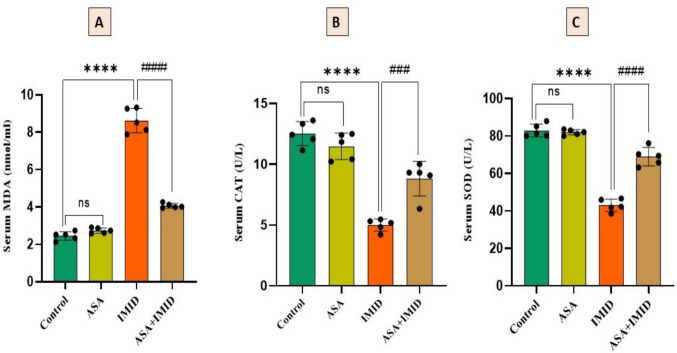
Impact of acetylsalicylic acid (ASA) against imidacloprid (IMID)-induced oxidative stress in liver. **A** MDA, **B** CAT, and **C** SOD. Values showed as mean ± SD (*n* = 5). Significant difference vs. *normal control (****p < 0.001), ^#^imidacloprid (.^####^*p* < 0.001)

Rats administered IMID showed a noteworthy increase in serum MDA level and decrease in CAT and SOD activities compared with control group (*p* < 0.001). Moreover, rats treated with ASA+IMID presented marked decrease in serum MDA level and CAT and SOD activities compared with IMID group (*p* < 0.001). Furthermore, no substantial difference was observed between the control and ASA groups (Figure 3).

### Histopathological examination of both hepatic and brain tissues

The liver of control animals demonstrated normal hepatic parenchyma of normal hepatocytes which arranged in plates from duplicate cords separated with sinusoids connected directly with the central vein. The other hepatic tissues including bile canaliculi and duct and blood vasculatures including hepatic veins and arteries were within normal. The liver of control and ASA groups showed normal limits without any pathological alterations. The liver of the IMID group showed features of hepatitis especially around the periportal area accompanied with marked mononuclear inflammatory cell infiltration mostly lymphocytes and macrophages. The hepatic blood vessels showed marked vascular congestion. The hepatocytes within the centrilobular and midzonal areas showed hepatic vacuolation. The liver of the ASA+IMID group showed marked decreased inflammatory features around the portal area and also the degenerative changes within the hepatocytes.

**Fig. 4:**
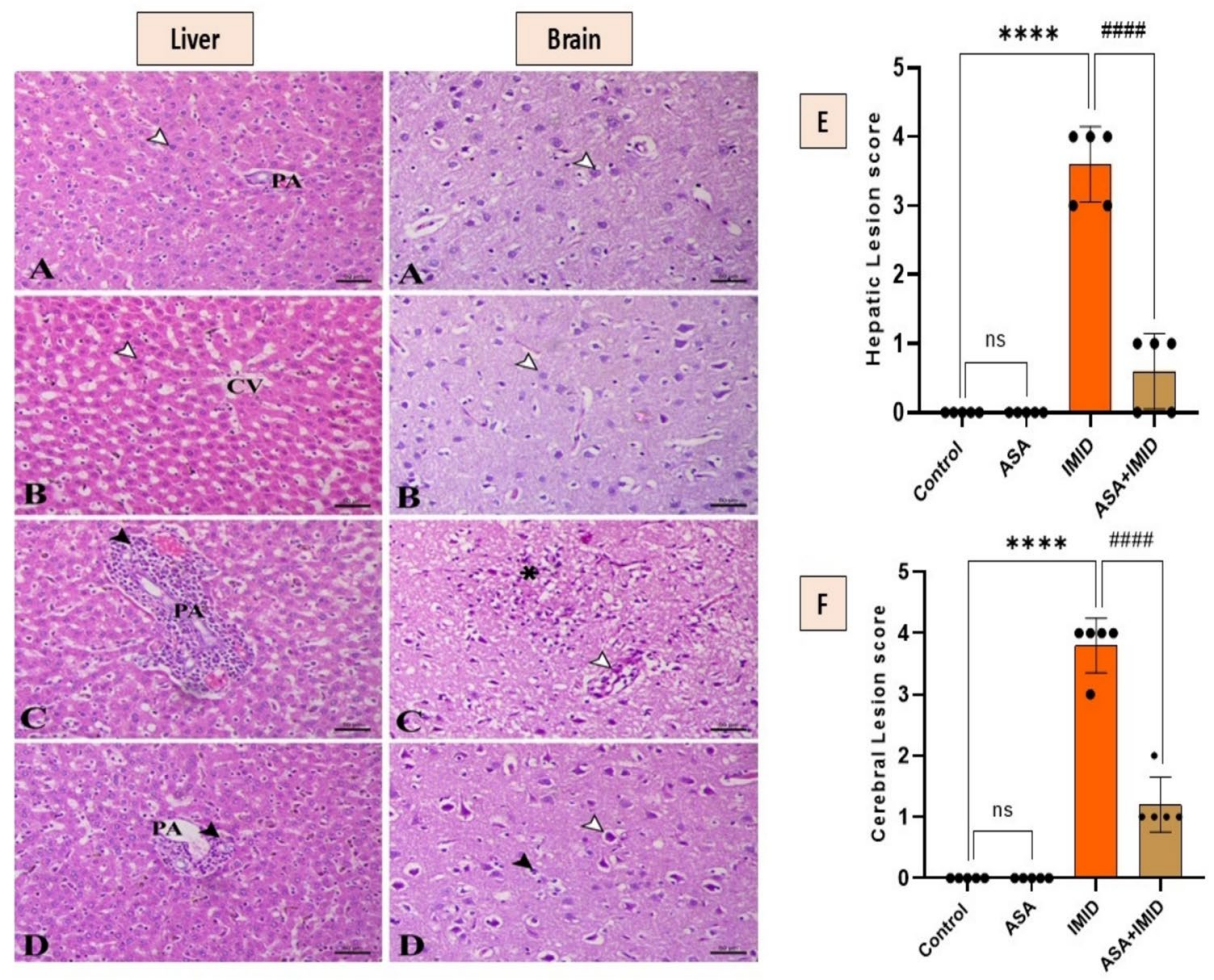
Hepatic sections of control and ASA groups (**A**, **B**, respectively) showing normal hepatic parenchyma (white arrowheads indicate normal hepatocytes and PA reveals portal area and CV indicates central vein). Hepatic section of IMID group showing portal inflammation (**C**, black arrowhead). Hepatic section of ASA + IMID group showing marked decrease in portal inflammation (**D**, black arrowhead). Brain sections of control and ASA groups (**A**, **B**, respectively) showing normal neuronal cells (white arrowheads). Brain section of IMID group showing focal area of malacia (asterisk) associated with injury to blood vessels (**C**, white arrowhead). Brain section of ASA + IMID group decrease malacic changes with marked decrease in portal inflammation (**D**, black arrowhead) and hepatic and brain lesions score, respectively (**E**, **F**). H&E stain, bar = 50 µm

The cerebral cortex of the brain of the control and ASA groups showed normal neurons embedded in a matrix of unmyelinated nerve fibers. Most of the neurons and fibers were within normal without any neuronal and/or axonal alterations. The brain of the IMID group showed multifocal cortical areas of malacia associated with disintegration of neuronal fibers and complete necrosis of the neuronal cells associated with microglia cells proliferation and activation. The blood vessels showed degenerative changes within their tunicae with perivascular lymphocytic cuffing. The brain of the ASA + IMID group showed marked decrease in the necrotic changes within the cerebral cortex associated with a noticeable decrease in gliosis. The neuronal cells showed ischemic features with a certain degree of neuronal vacuolation (Figure 4).

### The effect of acetylsalicylic acid (ASA) against imidacloprid (IMID)-reduced acetylcholinesterase activity (AchE) in the brain tissues

**Fig. 5:**
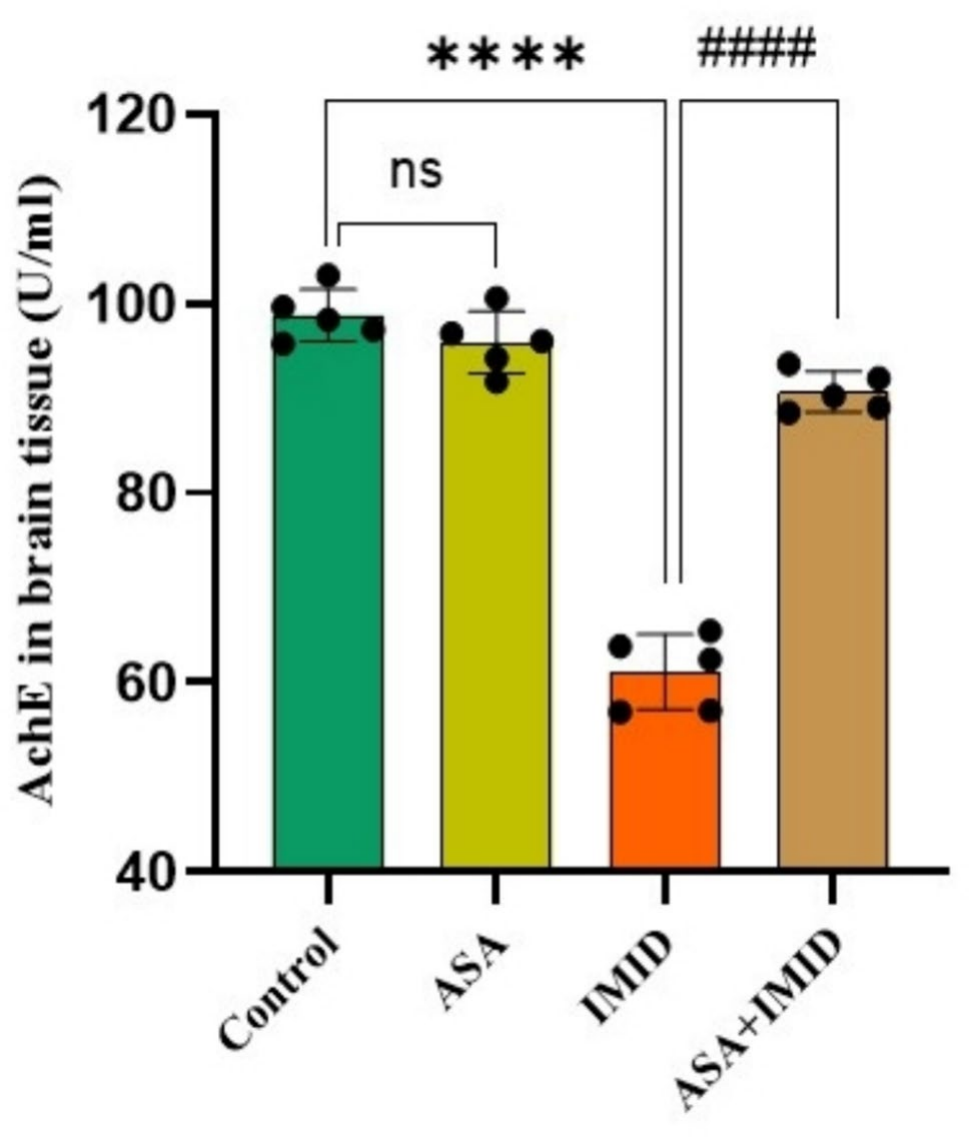
Impact of ASA and IMID on AchE enzyme activity in the brain tissues. Values showed as mean ± SD (*n* = 5). Significant difference vs. *normal control (****p < 0.001), ^#^imidacloprid (.^####^*p* < 0.001)

Rats treated with IMID exhibited a marked decrease in acetylcholinesterase enzyme (AchE) activity in brain tissues compared with control group (*P*< 0.001). Moreover, rats treated with ASA+IMID showed significant increase in AchE activity compared with IMID group (*p* < 0.001). Furthermore, no substantial difference was observed between the control and ASA groups (Figure 5).

### Immunohistochemical (IHC) analysis of NF-κB p65 in both liver and brain tissues

**Fig. 6:**
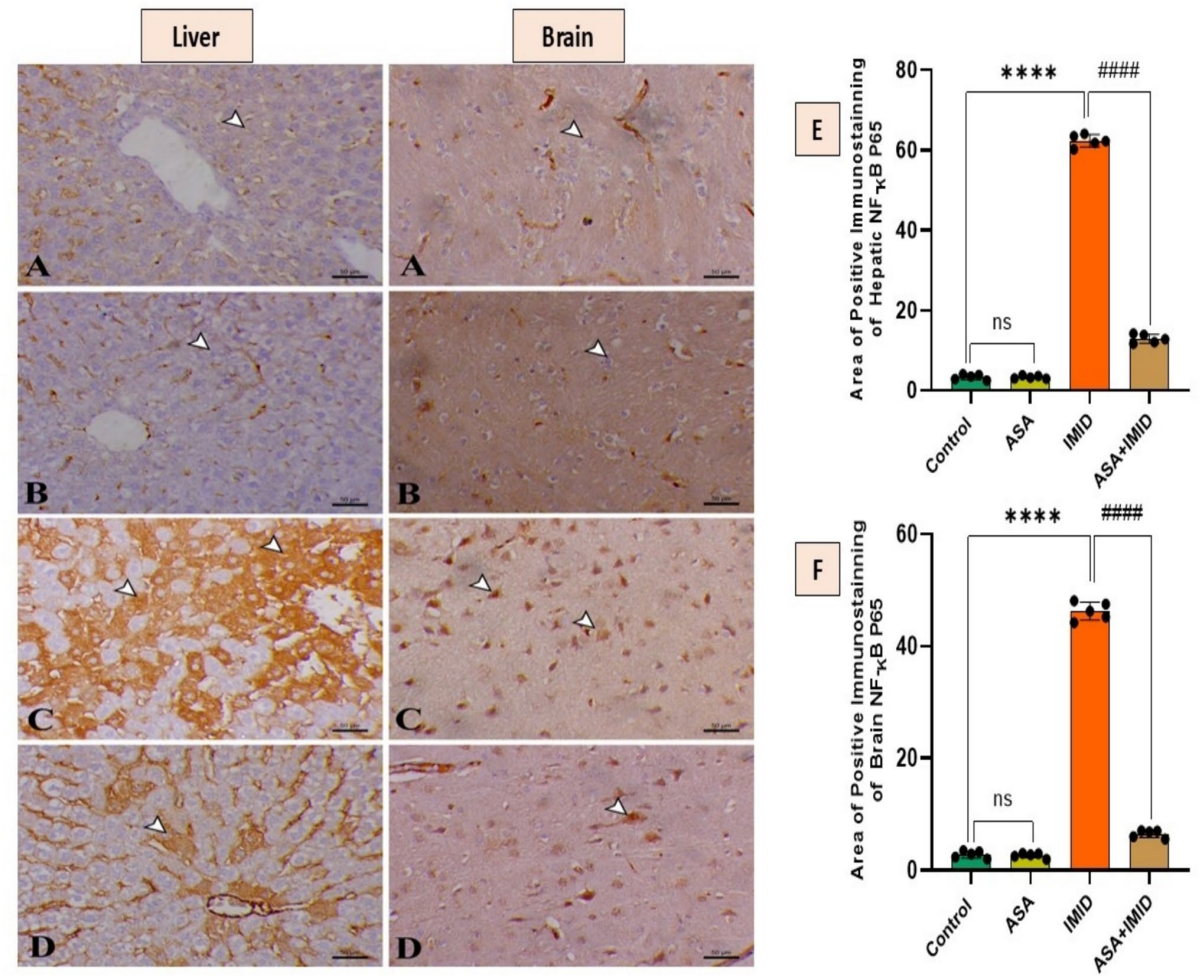
Hepatic and brain immunohistological sections of control and ASA groups (**A**, **B**, respectively), IMID group (**C**), ASA + IMID group (**D**), area of positive immunostaining of hepatic NF-κB P^65^ (**E**), and area of positive immunostaining of brain NF-κB P^65^ (**F**). Slight expression of NF-κB p65 antibody within the liver and brain tissues of control and ASA groups, with marked increase of the immunoexpression in both tissues in the IMID group, and marked decrease in intoxicated animals treated with ASA (white arrowheads reveal the positive immunostaining), NF-κB P65, bar = 50 µm

The expression of NF-κBP65 within hepatic and brain tissues showed minimal immunoexpression within the hepatic cells or neuronal cells either of control and groups. While the intoxicated animals with IMID showed a marked increase in cytoplasmic and nuclear expression within the hepatocytes and pyramidal neurons within the cerebral cortex, the animals of the ASA+IMID group showed a marked decrease in the positive cells within the parenchyma of the liver and brain tissues (Figure 6).

### The effect of acetylsalicylic acid (ASA) and imidacloprid (IMID) on the inflammatory mediators as tumor necrosis factor-alpha (TNF-α) and interleukin-6 (IL-6) in both liver and brain tissues

**Fig. 7:**
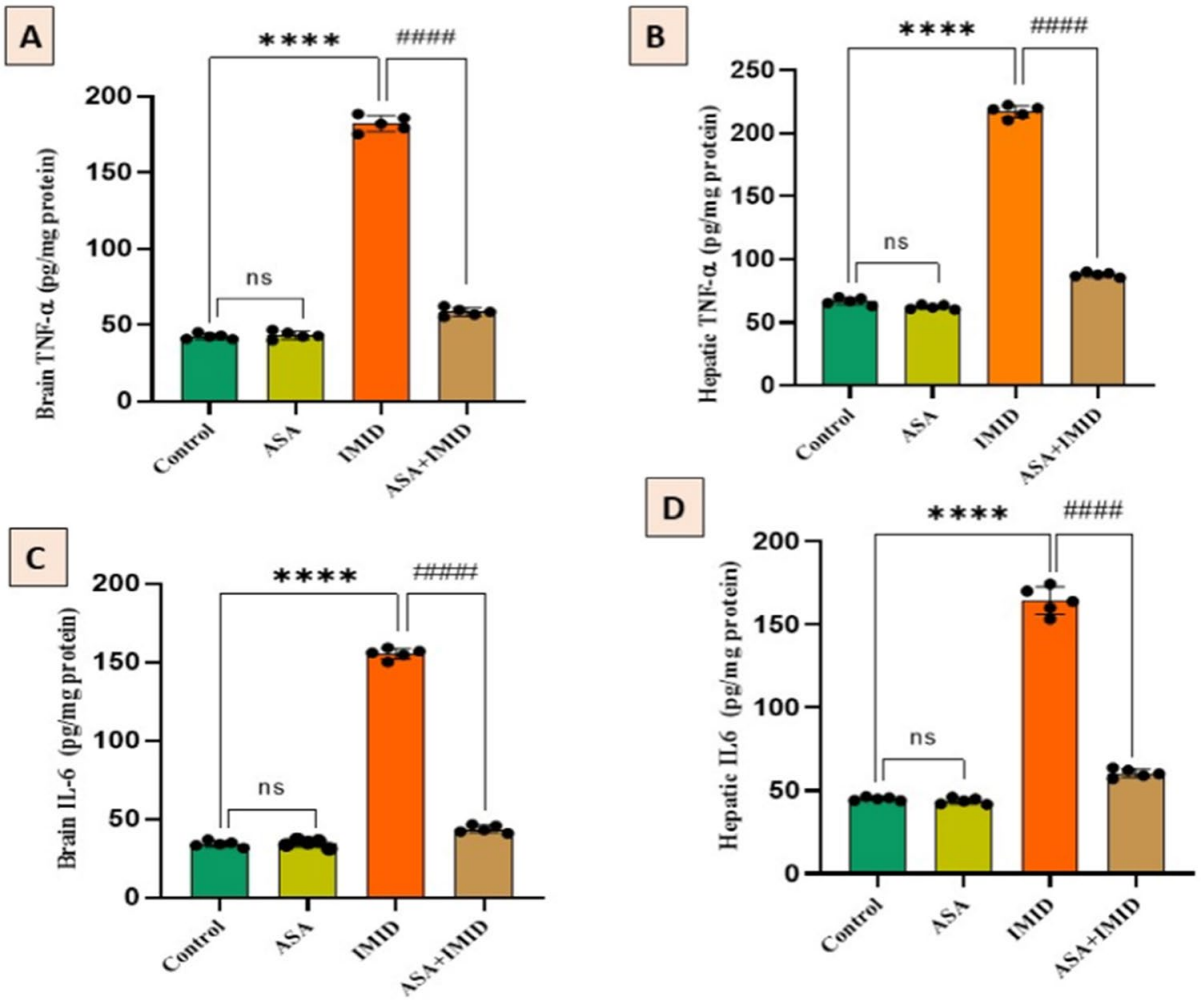
The impact of ASA and IMID on the inflammatory mediators as tumor necrosis factor-alpha (TNF-α) and interleukin-6 (IL-6) in both liver and brain tissues. **A** Brain TNF-α, **B** hepatic TNF-α, **C** brain IL-6, and **D** hepatic IL-6. Values showed as mean ± SD (*n* = 5). Significant difference vs. *normal control (****p < 0.001), ^#^imidacloprid (.^####^*p* < 0.001)

As shown in Figure 7, rats administered IMID displayed a noteworthy increase in TNF-α and IL-6 levels in both liver and brain tissues compared with control group (*p* < 0.001). Interestingly, rats treated with ASA+IMID showed noticeable decrease in TNF-α and IL-6 levels in both liver and brain tissues compared with IMID group (*p* < 0.001). Moreover, there is no significant difference between the control and ASA groups.

### Acetylsalicylic acid (ASA) suppressed mRNA expression of HMBG1, caspase-3, TNF-α, and IL-6 in imidacloprid (IMID) in both liver and brain tissues

**Fig. 8:**
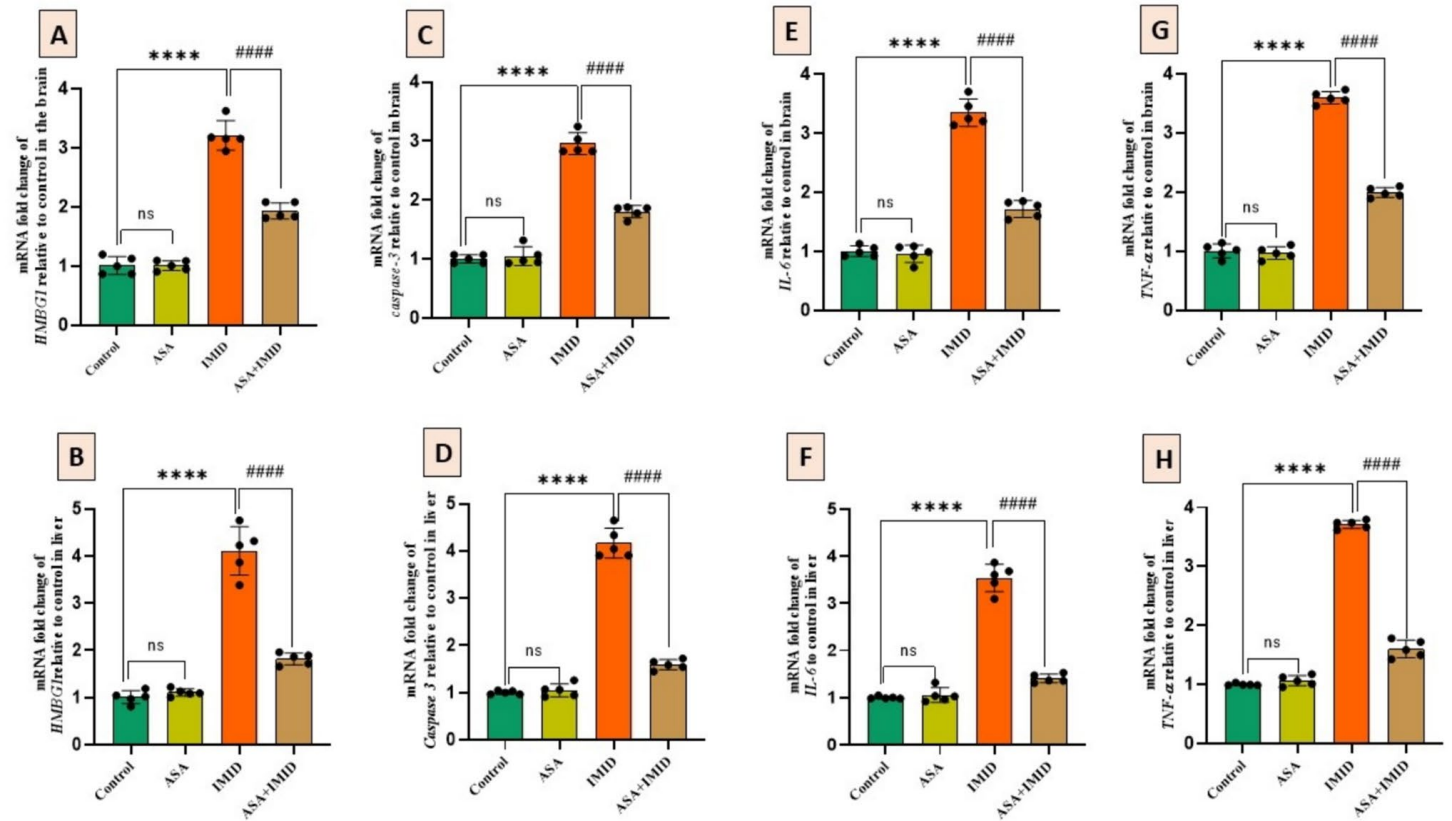
The impact of ASA and IMID on the mRNA of the HMBG1, caspase-3, tumor necrosis factor-alpha (TNF-α), and interleukin-6 (IL-6) relative mRNA expression of **A** brain HMBG1, **B** hepatic HMGB1, **C** brain caspase-3, **D** hepatic caspase-3, **E** brain TNF-α, **F** hepatic TNF-α, **G** brain IL-6, and **H** hepatic IL-6 in both liver and brain homogenates. Values showed as mean ± SD (*n* = 5). Significant difference vs. *normal control (****p < 0.001), ^#^imidacloprid (.^####^*p* < 0.001)

As shown in Figure 8, rats administered IMID exhibited a marked rise in the expression of HMBG1, caspase-3, TNF-α, and IL-6 in both liver and brain tissues compared with control group (*p* < 0.001). Remarkably, rats treated with ASA+IMID displayed noticeable decrease in the expression of HMBG1, caspase-3, TNF-α, and IL-6 in both liver and brain tissues compared with IMID group (*p* < 0.001).

## Discussion

Pesticide application is one of the primary methods for insect control in modern agriculture (Gruner et al. 2013; Sriapha et al. 2020). Among these, neonicotinoids are the most predominantly used and marketed insecticides globally, valued for their efficacy in eradicating pests (Mudgal et al. . 2023; Abd-Elhakim et al. 2018). However, significant concern has grown regarding the impact of neonicotinoids, particularly their role in the decline of non-target insects such as bees, which are vital pollinators. Consequently, the toxicity of pesticides is typically assessed through lab-based assays, though the empirical and theoretical foundations for extrapolating these results to field conditions remain markedly limited (Mahajan et al. 2018).

Imidacloprid (IMID) is a neonicotinoid insecticide that has been widely used for decades to control agricultural pests. It functions by disrupting normal neurochemical signal transmission in the central nervous system of insects (Pizzino et al. 2017). Due to its pervasive use, IMID has become a prevalent global contaminant and is known to have various detrimental effects on numerous non-target organisms, including animals and humans (Ge et al. 2015).

Consequently, the aim of our study was to explore the potential toxicological impact of acetylsalicylic acid (ASA) on the neonicotinoid insecticide imidacloprid (IMID) in animal model. While ASA is known for its anti-inflammatory and antithrombotic properties in medical contexts, its role in plant systems is distinct. In plants, salicylic acid is a functional compound of acts to enhance stress tolerance (Yang et al. 2023). Intriguingly, ASA was applied in combination with pesticides; it can reduce pesticide-induced phytotoxicity, predominantly by alleviating oxidative stress through the upregulation of antioxidant defenses (Spormann et al. 2019). Furthermore, ASA can exhibit a synergistic action with certain pesticides, not as a direct toxicant, but by priming the plant’s own immune responses, such as systemic acquired resistance (SAR), which includes enhanced antiviral activity (Gruner et al. 2013).

In the present study, IMID administration has exhibited a significant hepatic and brain toxicity. Liver toxicity was indicated by the marked increase of ALT and AST activities and decrease of albumin and total proteins levels in comparison with control group. Moreover, histopathological changes of liver showed features of hepatitis especially around the periportal area accompanied with marked mononuclear inflammatory cells infiltration mostly lymphocytes and macrophages. In the same line, many previous studies have stated that IMID has a deleterious effect on the liver as indicated by marked increase of liver enzymes (AST and ALT) and histopathological change of the hepatic architecture (Arfat et al. 2014; Sriapha et al. Feb. 2020; Zeng et al. 2024). In addition to IMID hepatic toxicity, IMID exerted a significant toxicity in the brain as indicated by cortical areas of malacia associated with microgliosis. The blood vessels showed degenerative changes within their tunicae with perivascular lymphocytic cuffing. These results are in agreement with other previous studies stated that IMID caused a neuronal damage, neuronal degeneration, demyelination, and congestion of blood vessels (Abdelhafez et al. 2023; Hassanen et al. 2023; Mudgal et al. . 2023). IMID induces significant oxidative stress in both the liver and brain, which is a central mechanism underlying its toxicity in these organs (Abd-Elhakim et al. Dec. 2018; Mahajan et al. 2018). Oxidative stress occurs when there is an imbalance between the generation and accumulation of reactive oxygen species (ROS) in cells and tissues and the biological system’s capacity to neutralize these reactive species (Pizzino et al. 2017).

In the present study, IMID administration in rats has showed marked increase in the oxidative stress levels. Interestingly, IMID exhibited a significant rise in in MDA level and decrease in SOD and CAT activities in comparison with control group. These results are in parallel with histopathological damage in both liver and brain and confirm the increased level of the oxidative stress. In this context, many previous studies agree with our results and confirmed that IMID increases the level of the oxidative stress (Ge et al. . 2015; Wei et al. . 2024).

Oxidative stress induces the release of HMGB1 and the activation of caspase-3, initiating a cascade that facilitates apoptosis and inflammation via the HMGB1/caspase-3 axis. The HMGB1/caspase-3 axis is a vital mechanism connecting apoptotic cell death to inflammatory responses through HMGB1 release, which is regulated by caspase-3–mediated mitochondrial and molecular processes. This axis has crucial roles in inflammation, vascular disease, neurodegeneration, and cancer progression (Yang et al. . 2024; Kim and Kang 2018; Chen et al. 2022). This axis is modulated by redox signaling, MAPK pathways, and cellular stress proteins, rendering it a vital target for therapeutics designed to mitigate oxidative damage and inflammation (Tang et al. 2011; Yang et al. 2025).

In the current study, IMID has showed a significant increase in mRNA expression of HMBG1 and caspase-3 and subsequently TNF-α and IL-6 inflammatory mediators. The activation of HMBG1 and caspase-3 plays an important role in the inflammation process in both liver and brain. As mentioned before, IMID has an obvious indirect role in inflammation process. Numerous prior research indicate that IMID elevates inflammatory mediators such as TNF-α, IL-6, and IL-1β primarily via oxidative stress and immune cell activation, hence exacerbating inflammatory damage in the brain, liver, and other tissues (Duzguner and Erdogan 2012; Cestonaro et al. 2023). Acetylcholinesterase (AChE) is a cholinergic enzyme predominantly located in postsynaptic neuromuscular junctions, particularly in muscles and nerves. It promptly degrades or hydrolyzes acetylcholine (ACh), a naturally occurring neurotransmitter, into acetic acid and choline (McHardy et al. 2017; Čadež et al. 2021). In the present study, IMID decreased AchE activity in the brain in comparison with control group. These results uncover the role of IMID is the induction of neurotoxicity. The reduction of AChE activity results in the accumulation of acetylcholine by which activates cholinergic receptor, prolonged neuronal signaling, and toxicity. As a conclusion, all data presented in the current study indicates the toxic effect of IMID on both liver and brain.

Interestingly, administration of ASA has attenuated the toxic effect of IMID on the liver and brain. ASA improved liver functions as indicated by the decrease of ALT and AST activities and increased albumin and total proteins levels in comparison with IMID group. Moreover, ASA has restored the normal architecture of liver and decreased the necrotic changes within the cerebral cortex associated with decrease noticeable decrease gliosis brain tissues. These results are in agreement with previous studies that declare the hepatoprotective and neuroprotective effect of ASA in other models as hepatocellular carcinoma and ischemic stroke in rats, respectively (Wang et al. 2023; Yan et al. 2013).

ASA has improved the antioxidant activity and restored the balance of oxidative stress levels. ASA ameliorated the oxidative stress induced by IMID administration, as indicated by significant reduction of MDA level and increased the activities of both SOD and CAT. Many previous studies have showed the antioxidant activities of ASA in experimental models such as myocardial ischemia-reperfusion and brain slices subjected to hypoxia in rats (Jorda et al. 2020; De La Cruz et al. 2004; Frydrychowski et al. 2022).

Moreover, ASA administration showed anti-inflammatory properties as indicated by the attenuation of IMID induced HMGB1 and caspase-3 mRNA over expression. Subsequently, ASA decreased the levels of the inflammatory mediators such as TNF-α, IL-6, and IL-1β compared with IMID group. Moreover, ASA decreased the immunostaining level of NF-κB P65 as compared with IMID group. Interestingly, ASA administration showed an important neuroprotective effect as indicated by the increased AChE activity as compared with the IMID group. Increased AChE activity leads to the decomposition of acetylcholine to choline and acetic acid and inhibits the neuronal signal and neurotoxity. In the same line, previous study has showed that ASA in low dose can increase the AChE activity in aging model induced in rats (Vergil Andrews et al. 2024).

**Fig. 9:**
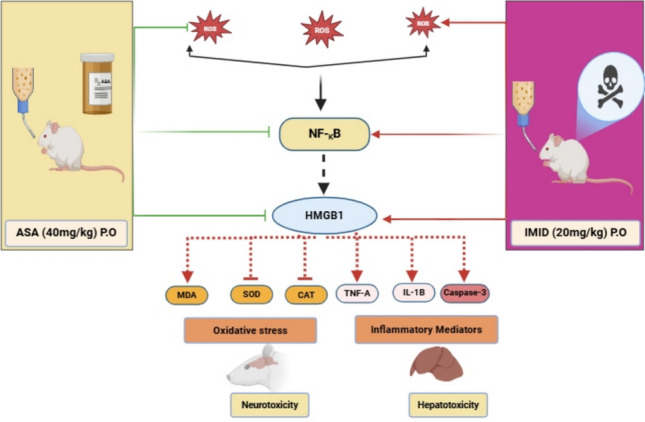
Schematic representation of acetylsalicylic acid (ASA) in the attenuation of imidacloprid (IMID)-induced neurotoxicity and hepatotoxicity in rats

Together, our results establish that ASA alleviates IMID-induced hepatotoxicity and neurotoxicity by inhibiting oxidative stress, inflammatory mediators as IL-1β and TNF-α, restoration of SOD and CAT activities, and inhibition of NF-κB/HMGB1/caspase-3 signaling pathway (Figure 9).

## Conclusion

In the current study, ASA demonstrated a promising protective effect against IMID-induced hepatotoxicity and neurotoxicity. IMID triggered cellular damage by depleting antioxidant stores and activating the HMGB1/caspase-3/NF-κB P65 axis, which resulted in a marked pro-inflammatory state, as assessed in both hepatic and cerebral tissues. Notably, ASA treatment restored oxidative balance and significantly reduced the necrosis and inflammation associated with IMID toxicity. In conclusion, our findings suggest the possible role of ASA in attenuating the toxicological effects of IMID in both the liver and the brain tissues.

## Data Availability

Data are available upon request.

## Abbreviations

Ach: Acetylcholine
AchE: Acetylcholinesterase
ALT: Alanine aminotransferase
ASA: Acetylsalicylic acid
AST: Aspartate aminotransferase
CAT: Catalase
GAPDH: Glyceraldehyde 3-phosphate dehydrogenase
HMGB1: High mobility group box 1
IL-6: Interleukin-6
IMID: Imidacloprid
MDA: Malondialdehyde
NF-κB: Nuclear factor kappa B
qRT-PCR: Quantitative real-time polymerase chain reaction
SOD: Superoxide dismutase
TNF-α: Tumor necrosis factor-alpha

## References

[CR1] Abdel razik MAA, Al Dhafar ZM, Alqahtani AM, Osman MA, Sweelam ME (2022) Dissipation and residues of imidacloprid and its efficacy against whitefly, *Bemisia tabaci*, in tomato plants under field conditions. Molecules 27(21):7607. 10.3390/molecules27217607 36364434 10.3390/molecules27217607 PMC9659103

[CR2] Abdelhafez HEDH, Hammam FM, El-Dahshan AA, Abodalam H, Guo J (2023) Imidacloprid induces neurotoxicity in albino male rats by inhibiting acetylcholinesterase activity, altering antioxidant status, and primary DNA damage. J Toxicol 1:4267469. 10.1155/2023/4267469 10.1155/2023/4267469 PMC10506876 37727350

[CR3] Abd-Elhakim YM, Mohammed HH, Mohamed WAM (2018) Imidacloprid impacts on neurobehavioral performance, oxidative stress, and apoptotic events in the brain of adolescent and adult rats. J Agric Food Chem 66(51):13513–13524. 10.1021/acs.jafc.8b05793 30501185 10.1021/acs.jafc.8b05793

[CR4] Architha M, Ravikumar Y, Chandravathi T, Anilkumar B, Aasritha S (2023) Haematological changes induced by imidacloprid (IMI) and chlorpyrifos (CPF) in male wistar rats. Pharma Innov J 12(1):1695–1698. Available: www.thepharmajournal.com. Accessed 22 Aug 2025

[CR5] Arfat Y et al (2014) Effect of imidacloprid on hepatotoxicity and nephrotoxicity in male albino mice. Toxicol Rep 1:554–561. 10.1016/j.toxrep.2014.08.004 28962268 10.1016/j.toxrep.2014.08.004 PMC5598541

[CR6] Bancroft JD, Gamble M (2007) Theory and practice of histological techniques, sixth edition. Theory Pract Histol Tech Sixth Ed 67(6):1–725. 10.1097/nen.0b013e31817e2933

[CR7] Čadež T, Kolić D, Šinko G, Kovarik Z (2021) Assessment of four organophosphorus pesticides as inhibitors of human acetylcholinesterase and butyrylcholinesterase. Sci Rep 11(1):1–11. 10.1038/s41598-021-00953-9 34728713 10.1038/s41598-021-00953-9 PMC8563940

[CR8] Cartereau A, Taillebois E, Le Questel JY, Thany SH (2021) Mode of action of neonicotinoid insecticides imidacloprid and thiacloprid to the cockroach pameα7 nicotinic acetylcholine receptor. Int J Mol Sci 22(18):9880. 10.3390/ijms22189880 34576043 10.3390/ijms22189880 PMC8471617

[CR9] Cestonaro LV et al (2023) Immunomodulatory effect of imidacloprid on macrophage RAW 264.7 cells. Environ Toxicol Pharmacol 101:104190. 10.1016/j.etap.2023.104190 37336278 10.1016/j.etap.2023.104190

[CR10] Chen R, Kang R, Tang D (2022) The mechanism of HMGB1 secretion and release. Exp Mol Med 54(2):91–102. 10.1038/s12276-022-00736-w 35217834 10.1038/s12276-022-00736-w PMC8894452

[CR11] Chi W, Chen H, Li F, Zhu Y, Yin W, Zhuo Y (2015) HMGB1 promotes the activation of NLRP3 and caspase-8 inflammasomes via NF-κB pathway in acute glaucoma. J Neuroinflammation 12(1):1–12. 10.1186/s12974-015-0360-2 10.1186/s12974-015-0360-2 PMC4518626 26224068

[CR12] de L. P. Biondo-Simões M, de Azevedo Pessini VC, Ichi CA, Robes RR, Ioshii S (2021) Acetylsalicylic acid (Aspirin®) and liver regeneration: experimental study in rats. Rev Col Bras Cir 48:e20213164. 10.1590/0100-6991e-20213164 34816883 10.1590/0100-6991e-20213164 PMC10683428

[CR13] De La Cruz JP, Guerrero A, González-Correa JA, Arrebola MM, Sánchez De La Cuesta F (2004) Antioxidant effect of acetylsalicylic and salicylic acid in rat brain slices subjected to hypoxia. J Neurosci Res 75(2):280–290. 10.1002/jnr.10851 14705149 10.1002/jnr.10851

[CR14] Devi NN et al (2024) Toxic effects of chlorpyrifos on biochemical composition, enzyme activity and gill surface ultrastructure of three species of small fishes from India. Environ Sci Pollut Res 1–16. 10.1007/s11356-024-35498-7 10.1007/s11356-024-35498-7 39547993

[CR15] Duzguner V, Erdogan S (2012) Chronic exposure to imidacloprid induces inflammation and oxidative stress in the liver & central nervous system of rats. Pestic Biochem Physiol 104(1):58–64. 10.1016/j.pestbp.2012.06.011

[CR16] Frydrychowski P et al (2022) Cardioprotective effect of acetylsalicylic acid in the myocardial ischemia-reperfusion model on oxidative stress markers levels in heart muscle and serum. Antioxidants 11(8):1432. 10.3390/antiox11081432 35892634 10.3390/antiox11081432 PMC9332077

[CR17] Ge W, Yan S, Wang J, Zhu L, Chen A, Wang J (2015) Oxidative stress and DNA damage induced by imidacloprid in zebrafish (Danio rerio). J Agric Food Chem 63(6):1856–1862. 10.1021/jf504895h 25607931 10.1021/jf504895h

[CR18] Gruner K, Griebel T, Návarová H, Attaran E, Zeier J (2013) Reprogramming of plants during systemic acquired resistance. Front Plant Sci 4(JUL):51698. 10.3389/fpls.2013.00252 10.3389/fpls.2013.00252 PMC3711057 23874348

[CR19] Hassanen EI et al (2023) Potential mechanisms of imidacloprid-induced neurotoxicity in adult rats with attempts on protection using Origanum majorana L. oil/extract: in vivo and in silico studies. ACS Omega 8(21):18491–18508. 10.1021/acsomega.2c08295 37273614 10.1021/acsomega.2c08295 PMC10233680

[CR20] Jorda A et al (2020) Action of low doses of aspirin in inflammation and oxidative stress induced by aβ1-42 on astrocytes in primary culture. Int J Med Sci 17(6):834–843. 10.7150/ijms.40959 32218705 10.7150/ijms.40959 PMC7085272

[CR21] Karolczak K, Kamysz W, Karafova A, Drzewoski J, Watala C (2013) Homocysteine is a novel risk factor for suboptimal response of blood platelets to acetylsalicylic acid in coronary artery disease: a randomized multicenter study. Pharmacol Res 74:7–22. 10.1016/j.phrs.2013.04.010 23665469 10.1016/j.phrs.2013.04.010

[CR22] Kim JE, Kang TC (2018) Differential roles of mitochondrial translocation of active caspase-3 and HMGB1 in neuronal death induced by status epilepticus. Front Cell Neurosci 12:405971. 10.3389/fncel.2018.00301 10.3389/fncel.2018.00301 PMC6133957 30233331

[CR23] Mahajan L, Verma PK, Raina R, Pankaj NK, Sood S, Singh M (2018) Alteration in thiols homeostasis, protein and lipid peroxidation in renal tissue following subacute oral exposure of imidacloprid and arsenic in Wistar rats. Toxicol Rep 5:1114–1119. 10.1016/j.toxrep.2018.11.003 30456172 10.1016/j.toxrep.2018.11.003 PMC6231080

[CR24] Mahajan L, Verma PK, Raina R, Sood S (2018) Toxic effects of imidacloprid combined with arsenic: oxidative stress in rat liver. Toxicol Ind Health 34(10):726–735. 10.1177/0748233718778993 30033815 10.1177/0748233718778993

[CR25] McHardy SF, Wang HYL, McCowen SV, Valdez MC (2017) Recent advances in acetylcholinesterase inhibitors and reactivators: an update on the patent literature (2012-2015). Expert Opin Ther Pat 27(4):455–476. 10.1080/13543776.2017.1272571 27967267 10.1080/13543776.2017.1272571

[CR26] Mudgal R, Sharma S, Singh S, Ravichandiran V (2023) The neuroprotective effect of ascorbic acid against imidacloprid-induced neurotoxicity and the role of HO-1 in mice. Front Neurol 14:1130575. 10.3389/fneur.2023.1130575 37153653 10.3389/fneur.2023.1130575 PMC10157196

[CR27] Pang S et al (2020) Insights into the toxicity and degradation mechanisms of imidacloprid via physicochemical and microbial approaches. Toxics 8(3):65. 10.3390/TOXICS8030065 32882955 10.3390/toxics8030065 PMC7560415

[CR28] Pizzino G et al (2017) Oxidative stress: harms and benefits for human health. Oxid Med Cell Longev 2017:8416763. 10.1155/2017/8416763 28819546 10.1155/2017/8416763 PMC5551541

[CR29] Sidlak AM, Spadaro A, Koyfman A, Long B (2025) Acute salicylate toxicity: a narrative review for emergency clinicians. Cureus. 10.7759/cureus.91505 41049912 10.7759/cureus.91505 PMC12491073

[CR30] Silva AM, Martins-Gomes C, Ferreira SS, Souto EB, Andreani T (2022) Molecular physicochemical properties of selected pesticides as predictive factors for oxidative stress and apoptosis-dependent cell death in Caco-2 and HepG2 cells. Int J Mol Sci 23(15):8107. 10.3390/ijms23158107 35897683 10.3390/ijms23158107 PMC9331544

[CR31] Spormann S, Soares C, Fidalgo F (2019) Salicylic acid alleviates glyphosate-induced oxidative stress in *Hordeum vulgare* L. J Environ Manage 241:226–234. 10.1016/j.jenvman.2019.04.035 31005000 10.1016/j.jenvman.2019.04.035

[CR32] Sriapha C et al (2020) Imidacloprid poisoning case series: potential for liver injury. Clin Toxicol 58(2):136–138. 10.1080/15563650.2019.1616091 10.1080/15563650.2019.1616091 31092066

[CR33] Tang D, Kang R, Zeh HJ, Lotze MT (2011) High-mobility group box 1, oxidative stress, and disease. Antioxid Redox Signal 14(7):1315–1335. 10.1089/ars.2010.3356 20969478 10.1089/ars.2010.3356 PMC3048826

[CR34] Tang Y, Zhan Y, Gao S, Li T, Xuan H (2025) Hepatotoxicity of imidacloprid in zebrafish and the alleviating role of 10-hydroxy-2-decenoi acid: insights into oxidative stress, inflammation, and gut microbiota. J Hazard Mater 494:138695. 10.1016/j.jhazmat.2025.138695 40412317 10.1016/j.jhazmat.2025.138695

[CR35] Thyssen J, Machemer L (1999) Imidacloprid: toxicology and metabolism. Nicotinoid Insectic Nicotinic Acetylcholine Recept 213–222. 10.1007/978-4-431-67933-2_9

[CR36] Tudi M et al (2021) Agriculture development, pesticide application and its impact on the environment. Int J Environ Res Public Health 18(3):1–24. 10.3390/ijerph18031112 10.3390/ijerph18031112 PMC7908628 33513796

[CR37] Vergil Andrews JF, Selvaraj DB, Bhavani Radhakrishnan A, Kandasamy M (2024) Low-dose aspirin increases olfactory sensitivity in association with enhanced neurogenesis and reduced activity of AChE in the experimental aging mice. Med Drug Discov 22:100191. 10.1016/j.medidd.2024.100191

[CR38] Wang Y et al (2023) Hepatoprotective effects of aspirin on diethylnitrosamine-induced hepatocellular carcinoma in rats by reducing inflammation levels and PD-L1 expression. Sci Rep 13(1):21362. 10.1038/s41598-023-48812-z 38049630 10.1038/s41598-023-48812-z PMC10695938

[CR39] Wei F, Cheng F, Li H, You J (2024) Imidacloprid affects human cells through mitochondrial dysfunction and oxidative stress. Sci Total Environ 951:175422. 10.1016/j.scitotenv.2024.175422 39128528 10.1016/j.scitotenv.2024.175422

[CR40] Yan BC et al (2013) Neuroprotective effect of a new synthetic aspirin-decursinol adduct in experimental animal models of ischemic stroke. PLoS ONE 8(9):e74886. 10.1371/journal.pone.0074886 24073226 10.1371/journal.pone.0074886 PMC3779249

[CR41] Yang H et al (2023) Uncovering the mechanisms of salicylic acid-mediated abiotic stress tolerance in horticultural crops. Front Plant Sci 14:1226041. 10.3389/fpls.2023.1226041 37701800 10.3389/fpls.2023.1226041 PMC10494719

[CR42] Yang H et al (2024) Caspase-3/gasdermin-E axis facilitates the progression of coronary artery calcification by inducing the release of high mobility group box protein 1. Int Immunopharmacol 127:111454. 10.1016/j.intimp.2023.111454 38159554 10.1016/j.intimp.2023.111454

[CR43] Yang L, Li X, Zhu X, Ge F, Wang Y (2025) Involvement of role of HMGB1-NLRP3 pathway in systemic disorders. Front Cell Dev Biol 13:1600596. 10.3389/fcell.2025.1600596 40688353 10.3389/fcell.2025.1600596 PMC12271189

[CR44] Zeng M, Shi M, Jian X, Dong L (2024) Treatment of an accident of imidacloprid poisoning. Front Pharmacol 15:1421437. 10.3389/fphar.2024.1421437 39114363 10.3389/fphar.2024.1421437 PMC11303190

